# High intensity interval training for older adults – from the laboratory towards a home setting: a co-creation study

**DOI:** 10.1186/s11556-026-00401-5

**Published:** 2026-01-22

**Authors:** Sofi Sandström, Jennifer Frankel, Nina Lindelöf, Mattias Hedlund, Erik Frykholm, Helena Fridberg, Erik Rosendahl, Carl-Johan Boraxbekk, Marlene Sandlund

**Affiliations:** 1 Department of Diagnostics and Intervention, Umeå University, Umeå, Sweden; 2 Department of Neurology, Copenhagen University Hospital Bispebjerg, Copenhagen, Denmark; 3 Institute for Clinical Medicine, Faculty of Medical and Health Sciences, University of Copenhagen, Denmark Institute of Sports Medicine Copenhagen (ISMC), Copenhagen, Denmark; 4 Department of Community Medicine and Rehabilitation, Umeå University, Umeå, Sweden

**Keywords:** Exercise, Co-creation, Implementation, Chair stand

## Abstract

**Background:**

Physical exercise can help prolong healthy aging, yet few options enable older adults to exercise at very high intensities at home. A previous gym-based supramaximal High Intensity interval Training (HIT) protocol on stationary bicycles has shown promising results. Core components were supramaximal interval intensity that could be systematically modulated (controlled, individualized, escalated, and de-escalated) and protocol safety. In this exploratory co-creation study with older adults, we aimed to adapt the supramaximal HIT protocol for potential future implementation in older adults’ home settings.

**Methods:**

Eleven older adults (6 females; ages 69–74) with prior supramaximal HIT experience participated in this two-phase co-creation study. In phase one, co-creators engaged in workshops to identify, explore, and discuss available home-based training modalities. In phase two, co-creators took part in lab tests wherein suitable modalities were tested and compared to stationary bicycling regarding acute physiological responses and safety during a supramaximal HIT session (10 × 10-second intervals with 50-second passive recovery). Results were continuously merged with the protocol’s core components.

**Results:**

Physiological and emotional reactions to HIT, potential exercise modalities, and necessary protocol adaptations were identified in phase one. When merged with core components three modalities - walking up steps, chair stand, and rubber band cross country double poling - were selected for phase two testing. Of these, chair stand elicited physiological responses most comparable to stationary bicycling, while fulfilling all core components.

**Conclusions:**

We adapted a researcher-supervised watt-controlled supramaximal HIT-protocol on stationary bicycles to a chair stand protocol with audio metronome-controlled intensity for potential implementation at home. This adaptation is the first step towards a safe and scalable implementation of a home-based supramaximal HIT program for older adults.

## Background

There is widespread support for physical exercise mitigating age-related diseases such as cardiovascular and neurological disorders [1–3], however many people fail to meet exercise recommendations [4], and inactivity increases with older age [5]. Finding effective time-efficient exercise methods is important to prolong healthy aging and prevent unnecessary disease and suffering [6]. Unfortunately, exercise recommendations often fail to include very high intensity training, despite support for its positive and time-efficient effects [7–10]. Contrary to common belief, performance on supramaximal intensities, i.e., very short and intense efforts at intensities above that produced at one’s maximal oxygen uptake, is often better preserved in older adults, while aerobic endurance performance declines earlier [11]. This type of supramaximal High Intensity interval Training (HIT) utilizes an individual’s anaerobic capacity, resulting in both anaerobic and aerobic gains, such as improved cardiorespiratory fitness, cardiovascular function, muscle strength [12], and muscle power [13], and seems extra effective for older people [14].

A supramaximal HIT protocol on stationary bicycles was previously developed and adapted for older adults over 65 years of age [14]. A randomized controlled trial (RCT) tested the protocol, comparing the effects of supramaximal HIT to Moderate Intensity Training (MIT) among older, non-exercising adults. The supramaximal HIT-protocol was considered promising regarding adherence and attendance, with more positive and fewer negative experiences compared to MIT [15]. The protocol showed similar- to superior effects on cardiorespiratory fitness, working memory, blood pressure, and lower limb strength, with no serious adverse events occurring [12, 13]. Furthermore, participants experienced supramaximal HIT as invigorating and enjoyable, challenging negative age stereotypes regarding what kinds of exercise an older person should conduct [16].

While the protocol results were promising, widespread implementation is limited, and the protocol needs to be adapted to fit end-users’ needs, including the opportunity to exercise at home. However, stationary bicycles are expensive, and older adults often have a low disposable income [17]. Other circumstances, such as personal preferences to exercise at home, limited ability to travel to a gym [18], and global pandemics [19] highlight the need for evidence-based home exercise programs, which can improve measures of fitness in older adults [20].

End-users should be involved in tailoring health interventions. One option is through co-creation research, wherein end-users actively participate in the development of a product. Together, researchers and the community collaborate to increase the impact of research [21]. Meanwhile, end-user preferences need to be balanced with protecting the intervention’s core components. Core components are functions, principles, and activities necessary to maintain an intervention’s outcomes [22].

The aim of this exploratory co-creation study was to, in a collaboration between researchers and a group of older adults with experience exercising supramaximal HIT, adapt a supramaximal HIT protocol to the home environment. Specifically, we first aimed to identify suitable alternative modalities to stationary bicycling for supramaximal HIT. Second, we aimed to compare the acute physiological responses of alternative exercise modalities compared to cycling during a supramaximal HIT session. The goal was to develop an adapted supramaximal HIT protocol in line with both co-creator preferences and the original protocol’s core components.

## Methods

### Study design

This exploratory co-creation study was conducted in collaboration with older adults and an interdisciplinary research team consisting of physiotherapists [initials blinded and replaced with letters M.S, E.R, M.H., E.F., H.F., N.L.), a neuroscientist (C-J. B), an engineer (J.F.), and a psychologist (S.S.). This study is a continuation from a previously conducted RCT which assessed the effects of a supramaximal HIT protocol adapted for older adults. The protocol consisted of 10 × 6-second cycle sprint intervals at a controlled supramaximal intensity, i.e., an external intensity (in watts) above that produced at one’s maximal aerobic capacity. The original protocol was conducted in groups in a gym-setting and was researcher-supervised. Core components to the original HIT protocol were defined as: (1) 10 intervals at a supramaximal intensity; (2) ability to systematically modulate intensity (i.e. start at a controlled, individualized, and tolerable level and progressively adjust upwards or downwards) (3) a safe protocol (free from Serious Adverse Events) [13]. When translated to this co-creation study, we therefore required all adaptations to fulfill these criteria, i.e., 10 intervals at an intensity eliciting similar physiological response to supramaximal HIT on a stationary bicycle, ability to systematically modulate intensity, and maintain a safe protocol free from accidents

**Fig. 1:**
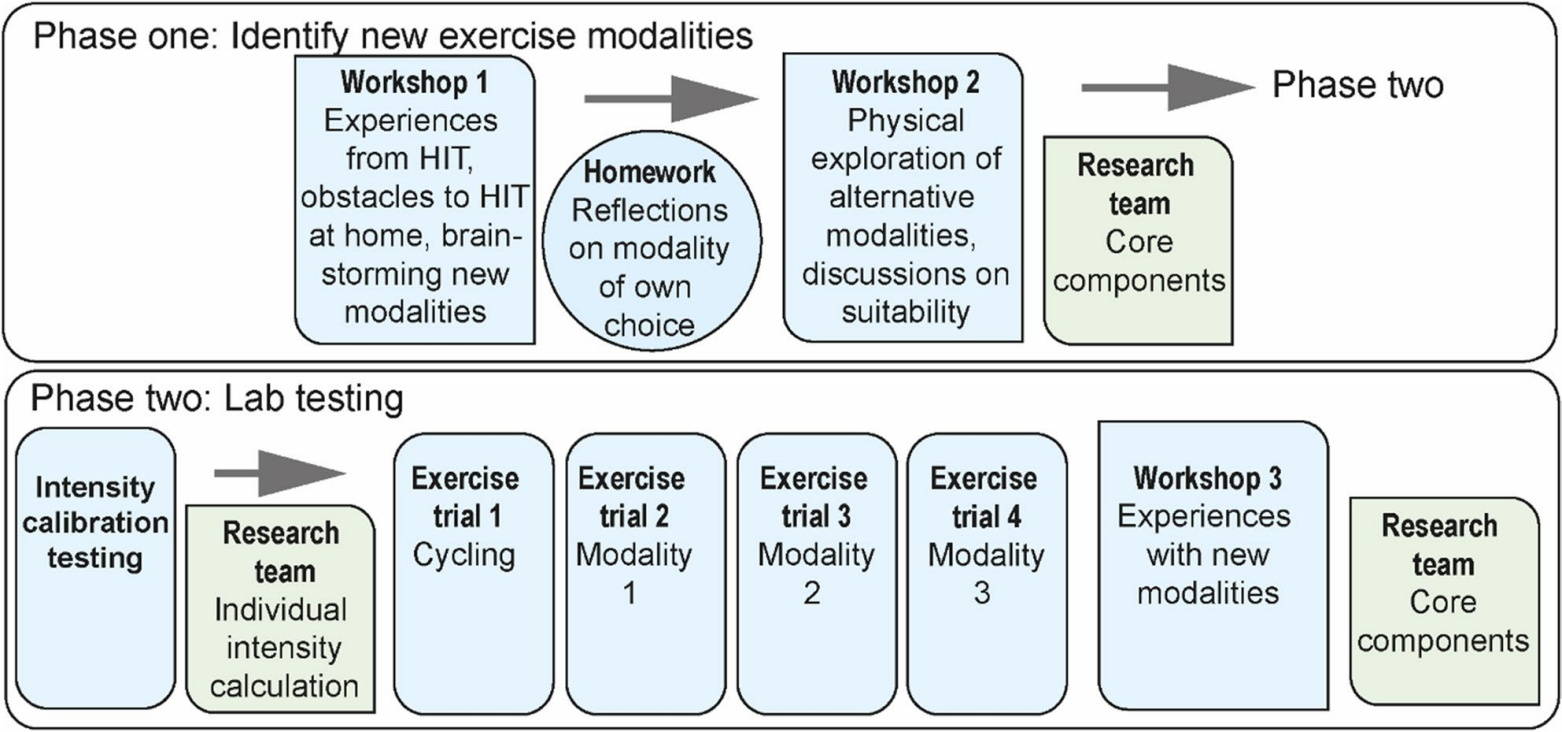
Study flow. Notes: Overview of phase one and two of the co-creation process. Blue indicates steps where co-creators worked in collaboration with the interdisciplinary researcher team. Green indicates steps where the interdisciplinary research team worked independently from co-creators

To adapt the original protocol to the home-setting of older adults, this co-creation study consisted of two phases, and co-creators took part in both, see Fig. 1 for study flow. Phase one, outlined below, consisted of co-creation workshops, conducted in-person in a group-format at the UMeHealth lab, a laboratory at Umeå University specifically designed to mimic a home environment. Workshops were facilitated by S.S., J.F., M.S., N.L., and H.F., and M.S., N.L., and H.F. had previous experience with co-creation and qualitative research. Prior to phase two, co-creators were medically screened by a cardiologist including a 12-lead electrocardiography, blood pressure measurements, and records of previous and current illness and medications. Phase two, also outlined below, was conducted in the Umeå Movement and EXercise lab (UMEX). Co-creators were brought into the lab individually and conducted one intensity calibration test and one supramaximal HIT exercise trial per exercise modality. Phase two test leaders M.H. and N.L. supervised laboratory testing and exercise science students helped gather data.

### Co-creators

Co-creators were defined as people with previous experience of exercising in the supramaximal HIT-group of the RCT, which was our inclusion criterion [12]. There were no exclusion criteria for phase one, however exclusion criteria for phase two were contraindications to high intensity training, such as untreated or poorly controlled hypertension, heart- or lung conditions with exercise-induced symptoms, movement-induced pain, or insulin-treated diabetes, based on the medical screening. All 34 individuals in the supramaximal HIT-group who had given their consent to be contacted after the RCT were contacted by mail with the aim of recruiting 10–15, based on similar participatory design studies [23].

### Data collection and analysis

#### Phase one

Phase one consisted of two workshops, with homework in between. Workshop one involved group discussions about co-creators’ memories of physiological and emotional responses elicited by the supramaximal HIT sessions during the RCT. Next, co-creators generated examples of everyday activities that elicit similar responses and discussed how suggested activities could translate to a supramaximal HIT-session. For homework, co-creators picked one modality and reflected upon how to perform it at home, potential obstacles, support needed, how to vary the intensity, and similarities and differences with HIT cycling.

Workshop two involved group discussions about the homework, followed by co-creators physically exploring several suggested modalities from workshop one and discussing their execution. There were four stations with 1–3 suggested modalities per station. A facilitator oversaw each station and asked questions about how to control and adjust intensity, ensure safety, and increase accessibility. Co-creators conducted a few repetitions of each modality at a low intensity, for example doing two slow lunges, and were instructed to skip any modality they felt uncomfortable with conducting.

During both workshops co-creators first reflected individually and wrote down their thoughts, followed by a discussion in small groups of 2–3 people, and ending with a larger group discussion. Throughout the process, facilitators reinforced that differing opinions were beneficial to the co-creation process.

The data collected during workshops consisted of audio recordings, facilitator- and co-creator notes, and photographs of whiteboard notes. Following each workshop two workshop facilitators (S.S. and J.F.) worked jointly, summarizing findings, which then were discussed with the other workshop facilitators to ensure that a shared understanding of the findings was achieved. Additionally, the content was reiterated with co-creators at the beginning of each workshop to ensure that the central aims of the workshops had not been overlooked or misunderstood. Data were sorted into the same content areas as the workshop themes; Physiological and emotional experiences when exercising supramaximal HIT, Potential exercise modalities, and Adjustments to exercising HIT at home. Each content area was summarized descriptively and illustrated with quotes.

#### Iterating phase one results with interdisciplinary research team

Between phase one and two, results were iterated within the interdisciplinary research team. Results from phase one were integrated with the core components. Discussions were focused upon whether the suggested modalities could be conducted as 10 supramaximal intervals and whether the intensity could be modulated. Co-creators’ experiences and views on safety were integrated with the workshop facilitators’ observations and the research team’s experiences of prescribing exercise to older adults.

#### Phase two

Exercise modalities selected from phase one were tested in the UMEX laboratory and compared to cycling. For each modality, one intensity calibration test and one exercise trial were conducted.

#####  Intensity calibration test

To determine individual interval intensity, a modality-specific calibration test was performed. For cycling, the Borg Cycle Strength Test [24] was used, where co-creators performed 30-second bouts at increasing resistance, with each bout separated by 30 s of rest, until reaching a Borg’s Rating of Perceived Exertion (RPE) 17. The highest completed level was the break point, considered to correspond to ~ 80% of 30-second maximal capacity. This model-based relationship was used to estimate 30-second maximal capacity, which in turn was assumed to represent ~ 70% of the maximal power attainable over 10 s, based on Wingate data in older adults [25] and analyses of data from Simonsson et al. [12], in line with standardized power-duration relationships. The procedure for setting brake force and cadence is described in detail in Simonsson et al., [12]. For non-cycling modalities, participants performed three 30-second trials of increasing self-selected effort (first easy, then moderate, and finally maximal), separated by two minutes of rest. The purpose of the two submaximal trials was to familiarize participants with the movement and ensure they felt comfortable performing an all-out effort on the final trial, which was the only trial used for intensity prescription. The number of repetitions during the maximal trial (max30) defined individual interval intensity, expressed as repetitions per minute. Test leaders also ranked safety on a 3-point scale: (1) able to perform the modality safely without supervision; (2) issues maintaining safety; and (3) unsafe performance of the modality (e.g., losing balance while performing).

Intensity calibration testing was conducted on two different days, with a minimum of four days of recovery in between. Each day included testing of two modalities in a non-randomized order with 30 min of rest in between.

##### Exercise trials

Each exercise trial consisted of a warmup followed by 10 × 10-second intervals interspersed with 50 s of passive recovery. Interval intensity was individualized and prescribed based on the calibration tests described above, using an audio metronome to control intensity. Each metronome beat corresponded to one movement (e.g., one step when climbing stairs) and guided co-creators in maintaining a high but controlled and individualized pace across all intervals. Exercise trials for each modality were conducted in the same order for all participants. There was a minimum of one day recovery in between exercise trials.

##### Outcomes

Outcomes of interest were Borg’s RPE, Borg’s Category Ratio 10 (CR10) for leg fatigue [24, 26], heart rate (HR), and the Feeling Scale (FS) [27], as well as test-leader ranked safety, as described above. Data was collected during several timepoints during each exercise trial and co-creators also rated their session average RPE following each exercise trial. Max HR and max RPE for each exercise trial were extracted. A recent review of subjective methods to determine exercise intensity concluded that RPE is appropriate to quantify the perceived effort at a given intensity, while CR10 is conceptually better suited to capture other symptoms, including local fatigue. The review also summarized the validity and reliability of both scales as high, independent of age, sex, exercise type, and activity level. FS was highlighted as an important measure of the affective response to exercise, which is a known moderator of future exercise behavior [28]. Together, these measures are regarded as reliable and appropriate for use in older adults and are commonly applied in exercise interventions.

Given that we previously found that the Borg Cycle Strength test correctly prescribed individualized supramaximal exercise intensities [12, 14] we inferred that exercise modalities eliciting similar RPE, CR10, and HR to supramaximal cycling illustrated similar supramaximal intensities. Due to a small sample size, no inferential statistical analyses were conducted, rather results were analyzed descriptively, reporting mean and standard deviations of peak exercise trial values. Furthermore, boxplots describe the distribution of data at several timepoints during the exercise trials to illustrate changes within a session. Analyses were done in in R [29] and RStudio [30] using the tidyverse package [31] and ggplot2 [32] for figures.

##### Qualitative feedback following testing

Following each exercise trial, co-creators filled out a written form with open-ended questions regarding their experiences of how the modality tested compared to exercise on a stationary bicycle. Following the completion of all exercise laboratory sessions, co-creators took part in a workshop during which they discussed their feedback on their exercise experiences, including similarities and differences between their experiences and the lab results. Data were summarized descriptively with the same procedure as phase one.

#### Iterating phase two results with interdisciplinary research team

Following phase two, results were iterated within the interdisciplinary research team to integrate findings with the core components. For core component 1 (10 supramaximal intervals) and core component 2 (systematic modulation of intensity) discussions focused on how non-cycling modalities compared to cycling regarding physiological responses during exercise trials. For core component 3 (safety), test leader safety rankings were evaluated.

## Results

Co-creator characteristics can be found in Table 1. Thirteen people expressed interest in participating and following a telephone call detailing the scope of the study, eleven agreed to join. One co-creator dropped out after participating in the first workshop during phase one, due to a misunderstanding that the study would entail regular exercise like the previous RCT. Three co-creators were excluded following medical screening, and the reasons for phase two exclusion were extensive heart disease (*n* = 1), untreated hypertension (*n* = 1), and movement-induced pain (*n* = 1). The remaining seven, described in Table 1, passed medical screening and continued to phase two.

**Table 1:** Co-creator characteristics for phase one and two

	Phase one (*n* = 11)	Phase two (*n* = 7)
Women, No. (%)	6 (54.6)	5 (71.4)
Age, years	70 (69, 74)	70 (69, 72)
Height, cm		167 (163, 177)
Body mass, kg		68.9 (48.3, 80.8)
BMI, kg/m^2^		23.7 (17.3, 30.0)
Blood pressure, mm Hg		
Systolic		126 (119, 140)
Diastolic		77 (74, 92)
Medical diagnoses, No. (%)		6 (85.7)
Diagnoses per person		2 (0, 4)
Medications, No. (%)		7 (100)
Medications per person		4 (1, 5)
BCST max, w		184 (148, 275)
Chair Stand 30s, n		25 (21, 33)

All values are expressed as median (min, max) unless otherwise specified. Descriptive health data was collected only for participants who proceeded to phase two. Medications reports number and percentage of participants with ≥ medications. Medications per person reports median number of medications and range across participants. Medical diagnoses reports number and percentage of participants with ≥ 1 medical diagnosis. Diagnoses per person reports median number of diagnoses and range across participants

*Abbreviations*: *BMI*  Body mass index, *BCST*  Borg Cycle Strength Test

### Phase one

Phase one findings are summarized in three categories: Physiological and emotional experiences when exercising supramaximal HIT; Potential exercise modalities; and Adjustments to exercising HIT at home.

#### Physiological and emotional experiences when exercising supramaximal HIT

Co-creators described certain physiological sensations as positive, such as being out of breath, feeling lactic acid in one’s legs, and being completely focused on the protocol.

*”It was exhausting, I was out of breath, and that means my heart and lungs were working. But it felt very nice afterwards, it passed quickly.”*

They appreciated that the session felt easy, due to the short duration of each interval, despite each individual interval being exhausting.

*”It* [the training session] *was incredibly easy*,* I was barely sweating. But that highest intensity*,* it was hard*,* you could barely complete it. But it was very short.”*

Co-creators also described negative experiences of feeling hand pain due to cycling being static, as well as feeling stressed out about correctly adjusting the brake resistance in such a short time.

*”But I also thought it was stressful*,* because you were trying to make the adjustments correctly. The only thing I was thinking about was to turn the knob at the right moment.”*

#### Potential exercise modalities

All suggested modalities are summarized in Table 2. For each modality, solutions to modulating intensity were discussed, such as adjusting speed, incline, size of movement, number of different body parts involved, or adding weight. For many modalities no solution to modulate intensity was found.

Co-creators agreed that the exercise modality should not be outdoors, or weather-, season-, or equipment-dependent, for availability purposes.

*”It* [the exercise modality] *should be easy to do. You shouldn’t need a lot of equipment. You should be able to do this with your everyday shoes and the clothes you wear inside.”*

Furthermore, they agreed that a suitable modality should work for a heterogenous group, including people with common physical limitations, such as sore knees or balance issues.

*”For me, I mean, I just don’t think most seniors maybe… To run is pretty hard, very straining, for knees, for joints, for your heart. I feel like, maybe the bar should be a bit lower?”*

This reflection was reinforced by facilitators’ workshop observations, where running, lunges, and step-up board caused balance issues for several co-creators.

**Table 2:** Suggested modalities

Suggested exercise modality	Comments on advantages and challenges
Jumping in place^†^ or with jump rope	Sore knees, hips, ankles. Balance issues.
Running^†^, walking, and Nordic Walking (uphill, treadmill, or in place)	Running is problematic due to sore knees, hips, ankles. Nordic Walking is weather- and season dependent. Walking in place is not high-intensive
Chair Stand^†^	Chair stand is promising.
Squats^†^, lunges^†^	Balance issues, especially lunges
Cross-country skiing / rubber band cross country double poling^†^	Cross-country skiing is weather-, season-, and equipment-dependent. Rubber band cross country double poling is promising.
Housework and yardwork	Not everyone has a yard. No solutions for intensity modulation.
Yoga, stretching, and toning	Not high-intensive.
Biking uphill	Season- and equipment-dependent.
Step-up board^†^, Walking up steps^†^	Step-up board caused balance issues during workshop. Walking up steps is promising.

All suggested modalities during phase one in left column

^†^ indicates the modality was physically explored during workshop two

#### Adjustments to exercising HIT at home

Co-creators expressed a need for alternatives to exercising on a stationary bicycle. They also discussed the structure of the training and adjustments that could increase user experience, such as help with keeping track of time, using an app, having a cue to push oneself, extending the intervals slightly (such as to 10 s), as well as a reminder to do an exercise session.

*”I think the problem is the timing. Because you get fixated upon it. One would need somebody on the side to tell you… Or help you… Or an app. I can’t both take the time and do the movement.”*

Many co-creators expressed a need for help reaching a high enough intensity when exercising on their own, as they had tried themselves at home without success. A metronome was suggested as a solution for this.

That is why you don’t see any results just from going on your walks. You don’t push yourself, and you don’t have anybody pushing you.

#### Integrating phase one co-creation with core components

Based on co-creator discussions, rubber band cross country double poling (home XC), chair stand, and walking up steps were considered promising and were iterated with the interdisciplinary research group, with discussions focused upon core components. All three suggested modalities were considered promising with the potential of eliciting supramaximal intensities that could be modulated with an audio metronome. Both co-creators and workshop facilitators considered the modalities to be safe, and the interdisciplinary research team had previous experience prescribing similar exercise modalities to older adults with success.

Other results from phase one that were in line with the core components and therefore applied in phase two were the use of an audio metronome to control intensity, extending the intervals to 10 s, and simplifying the protocol by making between-interval recovery passive.

### Phase two

**Fig. 2:**
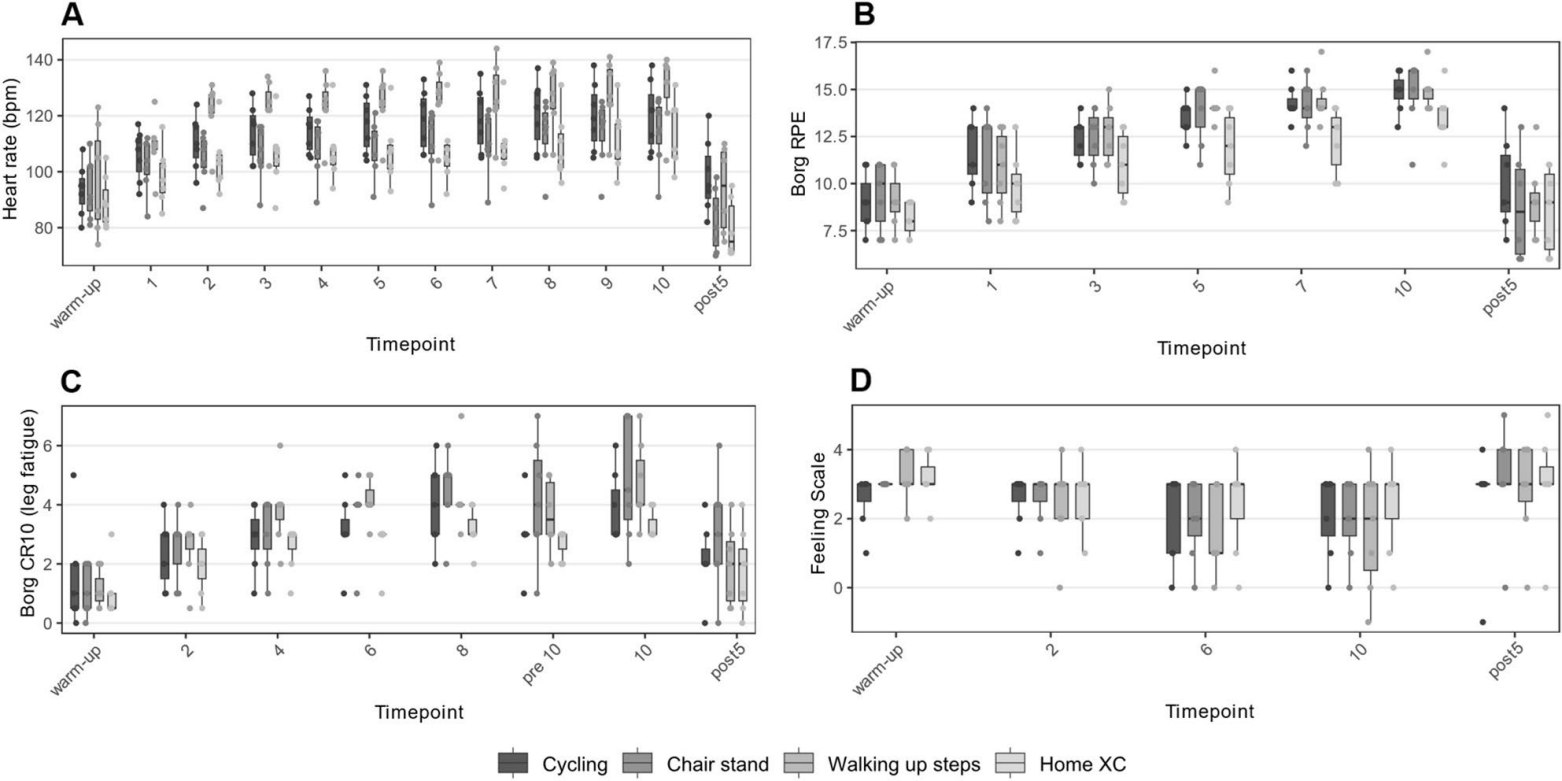
Acute physiological responses of potential training modalities. **A** Heart Rate response during warm-up, all 10 intervals, and five minutes post session for each modality. **B** Borgs Ratings of Perceived Exertion (scale 6–20) during warm-up, intervals 1, 3, 5, 7, and 10, as well as five minutes post session. **C** Leg fatigue, as measured with Borg CR10 scale (scale 0–10) at warm-up, interval 2, 4, 6, 8, rest period immediately preceding interval 10 (“pre 10”), during interval 10, as well as five minutes post session. **D** Feeling Scale (scale − 5 to + 5) response during warm-up, interval 2, 6, and 10, as well as five minutes post session

Chair Stand and walking up steps elicited interval-specific physiological responses comparable to cycling, including HR, RPE, CR10, and FS, see Fig. 2. However, max HR differed across modalities, see Table 3. In contrast, home XC elicited physiological responses inconsistent with supramaximal HIT on a stationary bicycle with regards to interval HR, interval RPE, and max RPE.

**Table 3:** Physiological responses for potential modalities and cycling

	Walking up steps	Chair stand	Home XC	Cycling
Max HR	130.7 (12.4)	114.7 (12.4)	114.3 (11.5)	121.7 (12.4)
Max RPE	15 (1)	15 (1.5)	13.4 (1.5)	14.9 (1.1)
Session RPE	14.3 (1.1)	14.1 (0.9)	12.7 (2.1)	13.7 (0.8)

Values are depicted as mean (sd)

#### Safety

While walking up steps physiologically resembled cycling, test leaders ranked level 2) during two incidents, with co-creators showing signs of problems maintaining safety when performing the modality, and several co-creators needed to slow down to maintain balance. Furthermore, a few co-creators required far more stairs to complete intensity calibration testing than was expected, and the availability of tall enough buildings became a limitation for widespread implementation.

#### Experiences exercising HIT on new modalities

Co-creators enjoyed all three modalities and discussed the pros and cons of their exercise experience.

For home XC, they appreciated using their upper body, however most co-creators agreed that home XC was less exhausting than cycling and presented issues with correctly adjusting the rubber band. Walking up steps elicited a strong physiological response, however co-creators pointed out that not everyone has staircases available, and that safety was an issue.

For Chair Stand, they enjoyed the simple instructions, the efficiency of the session, the fatigue in their legs, and the movement itself. However, almost all experienced muscle soreness the days following the exercise trial, which wasn’t a negative experience, although they believed future end-users should be forewarned of this side-effect.

*”I thought that* [Chair Stand] *was a nice movement. And then you get sweaty and a high heart rate and something happens in your whole body. My legs were completely gone*,* I was sore for three days.”*

Furthermore, co-creators appreciated the audio metronome and wanted it to be built into an app so that they didn’t need to keep track of timing or intensity parameters themselves. They wanted to be told what to do and when to do it.

#### Integrating phase two results with core components

Home XC was disqualified as a potential candidate due to exhibiting physiological responses inconsistent with supramaximal HIT on stationary bicycles, which was not in line with core component one. Furthermore, systematic intensity modulation proved problematic, due to technical difficulties being experienced by co-creators and observed by test leaders, thus not fulfilling core component two. Walking up steps was also disqualified as it was deemed unsafe, thus not fulfilling core component three. Meanwhile, chair stand showed similar physiological responses to supramaximal HIT on a stationary bicycle, intensity could be modulated, and was considered safe, fulfilling all three core components. Chair stand was therefore considered the best modality for future implementation.

## Discussion

Through this two-phase co-creation study, we adapted a researcher-supervised gym-based watt-controlled supramaximal HIT-protocol to a metronome-controlled supramaximal HIT-protocol for potential home implementation. We identified exercise modalities, obstacles, and solutions for protocol adaptation. We then tested the acute physiological responses of potentially suitable modalities. Chair stand was considered a viable option for end-users while maintaining the protocol’s core components.

This process brought us from stationary bicycling to chair stand. The adapted protocol intends to increase cardiorespiratory fitness similarly to the original supramaximal HIT protocol, however this shift in modality may also alter muscular adaptations. Cycling consists primarily of concentric muscle contractions, and while this is an effective way to exercise supramaximal HIT, leading to improvements in many age-sensitive outcomes [12, 13], high-speed chair stand may provide further benefits. This may be due to high-speed chair stand utilizing a fast switch between eccentric and concentric muscle contractions, which may increase explosive strength [33]. Eccentric forms of exercise have been shown beneficial for mitigating age-related decline in daily living activities [34]. This may heighten the neuromuscular adaptations compared to cycling, and the acute muscle soreness co-creators experienced may indicate myofibrillar remodeling [35]. While chair stand is promising, certain aspects will likely differ between the adapted and original protocol due to the modality shift. For example, the participant’s body mass will affect chair stand intensity more than with stationary bicycling, with heavier participants likely experiencing the chair stand modality as more exhausting. While we tried to adjust for differences in body size by modifying the height of the chair used, end-user’s body mass will likely affect the execution of the movement, and future steps in protocol development may need to consider making further adjustments, such as increasing the height of the chair for people with high BMIs. This may have been reflected in the increased variability in CR10 leg response in chair stand compared to stationary bicycling. Furthermore, with biking, more variables can be manipulated, such as the cadence, brake resistance, physical adjustments to the bike seat and handlebar settings, as well as the opportunity to conduct intervals at very low intensities, and chair stand does not offer as many opportunities for such adjustments. While the original supramaximal HIT protocol on stationary bicycles led to similar- to superior effects on lower limb strength compared to MIT [12, 13], it would be interesting to test the adapted chair stand protocol’s long-term effects on cardiorespiratory fitness and muscular strength. One study comparing the acute physiological responses of 60-second intervals of leg press and cycling found no difference between conditions regarding oxygen uptake, respiratory exchange ratio, blood lactate, and energy expenditure [36]. As chair stand and leg press use similar muscle groups, this finding is promising in relation to our results as it indicates the potential for long-term adaptations to cardiorespiratory fitness, however whether this applies to an older population needs tested in future efficacy trials. Furthermore, chair stand and leg press differ with regards to balance requirements, which may be an issue for some of our intended end-users. Meanwhile, exercise-increases to lower limb strength is associated with increased activity in the prefrontal cortex of the brain and increased working memory performance [37], as well as decreasing fall risk in older adults [38], suggesting further benefits of a chair stand supramaximal HIT-program.

One innovative adaptation was using an audio metronome to control intensity by anchoring it to an individual’s max30 cadence. This shift from watts [14] to metronome-controlled intensity allows for individualized training loads and progression over time. Considering the similarity in acute physiological responses between cycling and chair stand, it seems our intensity calibration test correctly prescribed a supramaximal intensity, despite the shift from watts to cadence, thus fulfilling our core components of a supramaximal and controlled intensity. We hope to confirm this in future feasibility studies. Metronome-paced exercise has previously been used in walking studies for patients with Parkinson’s [39], post-stroke [40], multiple sclerosis [41], veterans with unilateral transtibial amputation [42], as well as an at-home intervention for patients with chronic obstructive pulmonary disease [43], and for older adults conducting functional training [44], however to the best of our knowledge, this is the first study using it to individually adjust prescribed supramaximal HIT. While cadence achieved at max30 was used for intensity prescription, future implementation could adjust target cadence below max30 for beginners, allowing for more gradual progression, potentially mitigating muscle soreness.

The aim was not to conduct an in-depth qualitative analysis of the participants’ opinions and experiences, but rather to summarize their suggestions in an iterative process, which resulted in several adaptations to the protocol, including small modifications based on co-creators’ preferences. The intervals were extended from 6 to 10 s, and the between-interval recovery decreased from 54 to 50 s to maintain the pedagogical aspect of intervals starting once per minute. To decrease the number of adjustments during recovery, the adapted protocol instead implemented passive recovery. These changes are not considered large enough to impact the physiological benefits of the exercise [45], in line with the protocol’s core components.

Notably, descriptions such as feeling out of breath, fatigue in one’s legs during the intervals, and feeling one’s heart rate were present in both the phase one category *Physiological and emotional experiences exercising HIT* and the phase two category *Experiences exercising HIT on new modalities.* These findings, together with descriptive statistics on HR, perceived exertion, leg fatigue, and affective response support the similarity of chair stand and the original cycling protocol regarding both subjective descriptions and objective measures. Previous research indicates that supramaximal HIT on stationary bicycles elicits similar affective responses as moderate intensity training [15], with a median rating of 3 (range − 1 to 5), which is comparable to the present study. This is important, as concerns have been lifted regarding the risk of supramaximal HIT leading to negative affective responses [46]. However, neither a previously published RCT [15] or the current study found this to be the case. One difference between cycling and chair stand was the muscle soreness following the chair stand sessions, as discussed above. While co-creators viewed this soreness as positive, they suggested that future end-users should be forewarned, and that a lower starting intensity could be implemented.

The goal of these two phases of the study wasn’t to provide a platform for exercise delivery, however co-creators suggested a mobile app. There are many advantages to app-based exercise delivery, including modulating intensity, in line with the protocol’s core components. While several co-creators preferred group- and gym-based exercise, others preferred to exercise alone. During the past few years, the number of mobile exercise apps has increased, and research supports home-based training and home-exercise using digital devices for improving fitness and physical function while reducing fall risk and number of falls among older adults [20, 47, 48]. Research on older adults’ digital literacy has previously assumed this age group less capable [49], however the number of older adults using smart technology has increased, and was found to play an important role for older adults in isolation during the Covid-19 pandemic [50, 51]. While developing and testing app feasibility was outside the scope of this study, both are important for evaluating whether this adapted HIT protocol can be implemented in the home environment.

Given the study design and the sample size, these findings should not be viewed as evidence of intervention effectiveness. While we succeeded in recruiting 10–15 co-creators, our findings are limited due to the exclusion of several co-creators for phase two, indicating a potential barrier for future implementation. Exclusion was based on risk rather than medical diagnosis, resulting in a study sample that was relatively healthy, while also showing health-related variability, such as BMI ranging from underweight to obese. The question of how to adequately screen without exaggerated exclusion when implementing exercise interventions is an ongoing question in the field [52, 53]. This type of individualized exercise with built-in flexibility in intensity progression is promising for broader implementation, and an ongoing RCT using the same supramaximal HIT protocol has been tested on patients with chronic obstructive pulmonary disease, indicating that certain patient groups may also be potential end-users [54]. Another important aspect to consider when viewing our results is whether we are capturing “true” end-users, considering that our co-creators have registered for two exercise studies, indicating a high level of motivation [55, 56]. Bringing in broader perspectives and identifying structural and societal obstacles to implementation [57] is an important step when taking results from an RCT into the real world. Co-created interventions are context-specific, which limits direct generalizability. Meanwhile, participatory design ensures ecological validity and acceptability, while generating transferable insights that can inform adaptation in other settings.

With that said, this study also has limitations. We did not conduct a maximal cardiorespiratory fitness test, and therefore we do not have estimated maximum HR for our participants. Our results on HR are therefore expressed in absolute rather than relative terms, which increases the between-subject variability in the findings reported. Furthermore, interval prescription was based on an adapted calibration test, where we inferred that a similar relationship between 30-second maximal capacity corresponding to approximately 70% of the maximal power attainable over 10 s would hold for both stationary bicycling and alternative exercise modalities. We acknowledge there may have been better ways to prescribe intensity for other modalities, however we also note that our intensity prescription does seem to have been successful, given the similar acute physiological responses between chair stand and stationary bicycling during interval sessions. Finally, while implementation of home-based HIT is our long-term goal, we have not tested the protocol in the home environment, limiting our conclusions regarding whether the adapted HIT protocol can be implemented there. This will need testing in upcoming steps of the study.

This study is an example of how a lab-developed gym-based researcher-supervised supramaximal HIT protocol can be adapted for potential implementation in the home settings of older adults. Adaptation of results from exercise trials to real-life settings is important, as exercise can mitigate many age-related diseases that currently are increasing [1, 2] while helping older adults maintain a high quality of life and independence [58]. Future directions of this research involve developing a mobile app for exercise delivery, evaluating user experience, testing the adapted protocol’s feasibility, and evaluating long-term effects. Through this, we hope to increase the menu of efficient evidence-based exercise options for older adults.

## Conclusions

This exploratory two-phase co-creation study adapted a supramaximal HIT-protocol, going from exercise on a stationary bicycle to chair stand; from watt-controlled to metronome-controlled; and from a protocol that was researcher-supervised to one that has the potential of being delivered through a mobile app. The findings in this co-creation study are anchored to potential end-users’ own experiences and preferences. While the results need to be tested in a feasibility study for wider generalizability, this co-creation approach allows for important insights that traditional research designs often overlook. This adaptation is the first step towards a safe and scalable implementation of a home-based supramaximal HIT program for older adults.

## Data Availability

The dataset supporting the results in the current study are available from the corresponding author on reasonable request.

## Abbreviations

CR10: Borg’s Category Ratio 10
FS: Feeling Scale
HIT: High Intensity interval Training
Home XC: rubber band cross country double poling
HR: Heart Rate
Max30: Max cadence for 30 s
MIT: Moderate Intensity Training
RCT: Randomized Controlled Trial
RPE: Borg’s Ratings of Perceived Exertion
UMEX: Umeå Movement and EXercise laboratory
